# Supplementary material for: Developing a reporting guideline for artificial intelligence-centred diagnostic test accuracy studies: the STARD-AI protocol
Source: BMJ Open. 2021 Jun 28;11(6):e047709. doi: 10.1136/bmjopen-2020-047709 (PMC8240576; doi:10.1136/bmjopen-2020-047709)
Supplement: Author's manuscript [file bmjopen-2020-047709.draft_revisions.pdf]

BMJ Open

BMJ Open is committed to open peer review. As part of this commitment we make the peer review history of every article we publish publicly available.

When an article is published we post the peer reviewers' comments and the authors' responses online. We also post the versions of the paper that were used during peer review. These are the versions that the peer review comments apply to.

The versions of the paper that follow are the versions that were submitted during the peer review process. They are not the versions of record or the final published versions. They should not be cited or distributed as the published version of this manuscript.

BMJ Open is an open access journal and the full, final, typeset and author-corrected version of record of the manuscript is available on our site with no access controls, subscription charges or pay-per-view fees (http://bmjopen.bmj.com).

If you have any questions on BMJ Open's open peer review process please email info.bmjopen@bmj.com

BMJ Open

Developing a Reporting Guideline for Artificial Intelligence Centred Diagnostic Accuracy Studies: The STARD-AI Protocol

Journal:	*BMJ Open*
Manuscript ID	bmjopen-2020-047709
Article Type:	Protocol
Date Submitted by the Author:	06-Dec-2020
Complete List of Authors:	Sounderajah, Viknesh; Imperial College London, Department of Surgery and Cancer<br>Ashrafian, Hutan; Imperial College London, Department of Surgery and Cancer; Imperial College London, Department of Surgery and Cancer<br>Golub, Robert; Journal of the American Medical Association<br>Shetty, Shravya; Google Health<br>De Fauw, Jeffrey; DeepMind Technologies Ltd<br>Hooft, Lotty; University Medical Center Utrecht, University of Utrecht, Cochrane Netherlands<br>Moons, Karel; Julius Center for Health Sciences and Primary Care, Epidemiology<br>Collins, Gary; University of Oxford, Centre for Statistics in Medicine<br>Moher, David; Ottawa Hospital Research Institute, Ottawa Methods Centre<br>Bossuyt, Patrick M; Amsterdam University Medical Centres<br>Darzi, Ara; Imperial College London, Institute of Global Health Innovation<br>Karthikesalingam, Alan; Google Health<br>Denniston, Alastair; Queen Elizabeth Hospital Birmingham, UK<br>Mateen, Bilal Akhter; The Alan Turing Institute,<br>Ting, Daniel; Duke-NUS Medical School,<br>Treanor, Darren; University of Leeds<br>King, Dominic; Imperial College London, Centre for Health Policy<br>Greaves, Felix; Imperial College London, Department of Primary Care and Public Health<br>Godwin, Jonathan; DeepMind Technologies Ltd<br>Pearson-Stuttard, Jonathan; Imperial College London, PCPH<br>Harling, Leanne; Imperial College London, Department of Surgery and Cancer<br>McInnes, Matthew; University of Ottawa,<br>Rifai, Nader; Harvard Medical School,<br>Tomasev, Nenad; DeepMind Technologies Ltd<br>Normahani, Pasha; Imperial College London, Department of Surgery and Cancer<br>Aggarwal, Ravi; Imperial College London, Department of Surgery and Cancer<br>Markar, Sheraz; Imperial College London,<br>Vollmer, Sebastian; The Alan Turing Institute<br>Panch, Trishan

	Liu, Xiaoxuan; University of Birmingham<br>Whiting, Penny; University of Bristol, School of Social and Community Medicine
Keywords:	Protocols & guidelines < HEALTH SERVICES ADMINISTRATION & MANAGEMENT, Health informatics < BIOTECHNOLOGY & BIOINFORMATICS, Quality in health care < HEALTH SERVICES ADMINISTRATION & MANAGEMENT

SCHOLARONE™
Manuscripts

I, the Submitting Author has the right to grant and does grant on behalf of all authors of the Work (as defined in the below author licence), an exclusive licence and/or a non-exclusive licence for contributions from authors who are: i) UK Crown employees; ii) where BMJ has agreed a CC-BY licence shall apply, and/or iii) in accordance with the terms applicable for US Federal Government officers or employees acting as part of their official duties; on a worldwide, perpetual, irrevocable, royalty-free basis to BMJ Publishing Group Ltd ("BMJ") its licensees and where the relevant Journal is co-owned by BMJ to the co-owners of the Journal, to publish the Work in this journal and any other BMJ products and to exploit all rights, as set out in our licence.

The Submitting Author accepts and understands that any supply made under these terms is made by BMJ to the Submitting Author unless you are acting as an employee on behalf of your employer or a postgraduate student of an affiliated institution which is paying any applicable article publishing charge ("APC") for Open Access articles. Where the Submitting Author wishes to make the Work available on an Open Access basis (and intends to pay the relevant APC), the terms of reuse of such Open Access shall be governed by a Creative Commons licence – details of these licences and which Creative Commons licence will apply to this Work are set out in our licence referred to above.

Other than as permitted in any relevant BMJ Author's Self Archiving Policies, I confirm this Work has not been accepted for publication elsewhere, is not being considered for publication elsewhere and does not duplicate material already published. I confirm all authors consent to publication of this Work and authorise the granting of this licence.

For peer review only

Developing a Reporting Guideline for Artificial Intelligence Centred Diagnostic Accuracy Studies: The STARD-AI Protocol

Authors:

Viknesh Sounderajah[1,2], Hutan Ashrafian[1,2], Robert Golub[11], Shravya Shetty[6], Jeffrey De Fauw[3], Lotty Hooft[18], Carl Moons[18], Gary Collins[17], David Moher[12], Patrick Bossuyt[13] and Ara Darzi[1,2] on behalf of the STARD-AI Steering Committee (Alan Karthikesalingam[6], Alastair Denniston[4,15,16], Bilal Mateen[18], Daniel Ting[10], Darren Treanor[20], Dominic King[21], Felix Greaves[5], Jonathan Godwin[3], Jonathan Pearson-Stuttard[9], Leanne Harling[2], Matthew McInnes[7], Nader Rifai[22], Nenad Tomasev[3], Pasha Normahani[2], Penny Whiting[23], Ravi Aggarwal[1], Sebastian Vollmer[19], Sheraz Markar[2], Trishan Panch[8] and Xiaoxuan Liu[4,15,16])

Author Affiliations

[1] Institute of Global Health Innovation, Imperial College London, United Kingdom

[2] Department of Surgery and Cancer, Imperial College London, United Kingdom

[3] DeepMind, United Kingdom

[4] Institute of Inflammation and Ageing, College of Medical and Dental Sciences, University of Birmingham, United Kingdom

[5] The National Institute for Health and Care Excellence, United Kingdom

[6] Google Health

[7] Department of Radiology, University of Ottawa, Canada

[8] Division of Health Policy and Management, Harvard T.H. Chan School of Public Health, United States of America

[9] School of Public Health, Imperial College London, United Kingdom

[10] Singapore Eye Research Institute, Singapore National Eye Center, Singapore

[11] JAMA (Journal of the American Medical Association), United States of America

For peer review only

[12] Ottawa Hospital Research Institute, Canada

[13] Department of Clinical Epidemiology, Biostatistics and Bioinformatics, University of Amsterdam, The Netherlands

[14] University Hospitals Birmingham NHS Foundation Trust, Birmingham, United Kingdom

[15] Health Data Research UK, London, United Kingdom

[16] Clinical Epidemiology Program, Ottawa Hospital Research Institute, Canada

[17]Centre for Statistics in Medicine, Nuffield Department of Orthopaedics, Rheumatology and Musculoskeletal Sciences, University of Oxford, Oxford OX3 7LD, United Kingdom

[18]Julius Center for Health Sciences and Primary Care, and Cochrane Netherlands, University Medical Center Utrecht, Utrecht University, Utrecht, The Netherlands

[19] Alan Turing Institute, Kings Cross, United Kingdom

[20] Leeds Teaching Hospitals NHS Trust, University of Leeds, Leeds, United Kingdom

[21] Optum, Paddington, London, United Kingdom

[22] Department of Laboratory Medicine, Boston Children's Hospital, Harvard Medical School, Boston, Massachusetts, United States of America

[23] School of Social and Community Medicine, University of Bristol, Bristol, United Kingdom

Author disclosures:

The views and opinions expressed herein are those of the authors and do not necessarily reflect the views of their employers or funders.

Corresponding author:

Mr Hutan Ashrafian BSc (Hons) MBBS MRCS PhD MBA

Institute of Global Health Innovation, 10th Floor, Queen Elizabeth Queen Mother building, St Mary's Hospital Campus, Praed Street, London, United Kingdom, W2 1NY

Telephone Number: +447799871597

For peer review only

E-mail: hutan@imperial.ac.uk

Funding:

Infrastructure support for this research was provided by the NIHR Imperial Biomedical Research Centre (BRC).

GSC is supported by the NIHR Biomedical Research Centre, Oxford, and Cancer Research UK (programme grant: C49297/A27294).

DT is funded by National Pathology Imaging Co-operative, NPIC (Project no. 104687) is supported by a £50m investment from the Data to Early Diagnosis and Precision Medicine strand of the government's Industrial Strategy Challenge Fund, managed and delivered by UK Research and Innovation (UKRI).

FG is supported by the National Institute for Health Research Applied Research Collaboration Northwest London

Data Statement:

There is no data in this work.

Word count (main body):

3201

Study Status:

Stage 2 of this study has been completed. Stage 3 (the modified Delphi consensus process) is underway.

For peer review only

Abstract

Introduction:

STARD was developed to improve the completeness and transparency of reporting in studies investigating diagnostic accuracy. However, its current form, STARD 2015 does not address the unique issues and challenges raised by artificial intelligence (AI) centred interventions. As such, we propose an AI-specific version of the STARD checklist (STARD-AI 2021), which focuses upon the reporting of AI diagnostic accuracy studies. This paper describes the processes and methods that will be used to develop STARD-AI.

Methods and analysis:

Following guidance from the EQUATOR network, the development of the STARD-AI 2021 checklist can be distilled into six stages. (1) A project organisation phase has been undertaken, during which a Project Team and a Steering Committee were established. (2) An item generation process has been completed following a literature review, a patient and public involvement and engagement (PPIE) exercise and an online scoping survey of international experts. (3) A three-round modified Delphi consensus methodology is proposed, which will culminate in a teleconference consensus meeting of experts. (4) Thereafter, the Project Team will draft the initial STARD-AI checklist and the accompanying statement. (5) A piloting phase amongst expert and non-expert users will be carried out to identify items which are considered to be unclear, ambiguous or missing. This process, consisting of surveys and interviews, will contribute towards the explanation and elaboration document. (6) Upon finalisation of the manuscripts, a further teleconference meeting between the Project Team and Steering Committee is proposed prior to dissemination and implementation.

Ethics and dissemination:

Ethical approval has been granted by the Joint Research Compliance Office at Imperial College London (SETREC reference number: 19IC5679). A tailored dissemination strategy will be aimed towards 5

groups of stakeholders: (a) academia, (b) policy, (c) guidelines and regulation, (d) industry and (e) public and non-specific stakeholders. We anticipate that dissemination will take place in Q2 of 2021.

Key words:

Diagnostic accuracy, reporting guideline, artificial intelligence, STARD, transparency

Word count: 300/300

For peer review only

Article Summary

Strengths and limitations of this study:

- Gap: There are no specific reporting standards for artificial intelligence (AI) diagnostic accuracy studies
- Solution: We are developing a specific set of reporting standards for AI diagnostic accuracy studies; STARD-AI 2021.
- Clinical implications: This will help key stakeholders to appraise quality and compare diagnostic accuracy of AI models that are reported scientific studies.
- Strengths: STARD-AI 2021 will be a product of extensive evidence generation process that is led by multiple stakeholders (clinician scientists, computer scientists, journal editors, EQUATOR Network representatives, reporting guideline developers, epidemiologists, statisticians, industry leaders, funders, health policy makers, patients, legal experts and medical ethicists).
- Limitations: views of Delphi panellists may differ from those experts who decline participation.

only

Glossary

Project Team

This consists of the founder of STARD (PMB), the former United Kingdom Minister for Health and the current chair for the National Health Service Accelerated Access Collaborative (AD), members of the TRIPOD-AI group (GSC, LH, KGM), a senior software engineer (SS), directors of the EQUATOR Network (DM, GSC), the scientific content deputy editor for JAMA (RG) as well as 2 clinician scientists from Imperial College London (HA (supervisor), VS (doctoral research fellow)).

Steering Committee

This consists of clinician scientists, computer scientists, journal editors, EQUATOR Network representatives, epidemiologists, statisticians, industry leaders, funders, health policy makers, legal experts and medical ethicists. These individuals were identified through their notable work with respect to (1) diagnostic accuracy research, (2) artificial intelligence in healthcare, (3) health policy, (4) contribution to AI-centred EQUATOR initiatives, such as TRIPOD-AI, CONSORT-AI and SPIRIT-AI.

Consensus Group

This consists of experts who participated in the modified Delphi consensus process of the study.

Pilot Group

This consists of experts who participated in the pilot phase (Stage 5) of the study.

Checklist

A document listing the minimally essential items that should be reported in all diagnostic accuracy studies centred around artificial intelligence interventions. This constitutes the core of the reporting guideline.

For peer review only

Statement

Provides the rationale in the development of this reporting guideline, describes the process of developing the checklist, the checklist, dissemination and implementation plans, and any evaluation plans.

Explanation and Elaboration (E&E)

Provides the rationale behind each item in the checklist, along with examples of good reporting.

Reporting guideline

The combination of the checklist, statement and E&E material.

Flow diagram

A flow diagram depicts the flow of information through the different phases of a study.

Artificial Intelligence (AI)

The science of developing computer systems which can perform tasks normally requiring human intelligence.

Delphi study

A research method that derives the collective opinions of a group through a staged consultation of surveys, questionnaires, or interviews, with an aim to reach consensus at the end.

For peer review only

Introduction

Artificial intelligence (AI) is commonly cited as an imminent disruptive innovation[1] within the health sector. If used successfully, AI has the potential to tackle (1) the high rate of avoidable medical errors, (2) workflow inefficiencies and (3) delivery inefficiencies associated with modern healthcare provision[2]. The majority of AI interventions that are close to translation are in the field of medical diagnostics[3]. In the current paradigm, diagnostic investigations require timely interpretation from an expert clinician in order to generate a diagnosis and to subsequently direct episodes of care. However, the recurring issue with the present system is that diagnostic services are inundated with large volumes of work, which often exceeds workforce capacity[4]; COVID-19 being an immediate case in point. In order to address this, diagnostic AI algorithms have positioned themselves as medical devices that may achieve diagnostic accuracy comparable to that of an expert clinician whilst concurrently alleviating health-resource use. Although this paradigm shift may seem imminent, it is crucial to note that much of the evidence supporting diagnostic algorithms has been disseminated in the absence of AI-specific reporting guidelines. Without this guidance, and in a relatively nascent area, key stakeholders are poorly placed to appraise quality and compare diagnostic accuracy between scientific studies.

The STARD (Standards for Reporting of Diagnostic Accuracy Studies) 2015 statement remains the most widely accepted set of reporting standards for diagnostic accuracy studies[5]. STARD was developed to improve the completeness and transparency of studies investigating diagnostic accuracy. It consists of a checklist of 30 items that authors are strongly encouraged to address when reporting their diagnostic accuracy studies. It is endorsed by over 200 biomedical journals[6] and studies have shown that adherence to the STARD checklist leads to improved reporting of key study parameters[7,8].

However, in its current iteration, STARD 2015 is not designed to address the issues and challenges raised by AI-driven modalities. Issues include unclear methodological interpretation (e.g., the use of

external validation datasets, complexities of datasets and comparison to human performance), the lack of standardized nomenclature (e.g., the definition of a 'validation dataset'), as well as the heterogeneity of outcome measures (e.g., area under the receiver operating characteristics (AUROC), sensitivity, positive predictive value and F1 score). Until these issues are overcome, achieving comprehensive evaluations of these technologies and their potential translational benefits will remain limited.

In order to tackle these problems, we propose an AI-specific STARD guideline (STARD-AI) that aims to focus upon the reporting of AI diagnostic accuracy studies[9]. This work is complementary to the other AI centred checklists listed in the EQUATOR (Enhancing Quality and Transparency of Health Research) Network program (www.equator-network.org)[10], such as SPIRIT-AI (Standard Protocol Items: Recommendations for Interventional Trials)[11], CONSORT-AI (Consolidated Standards of Reporting Trials)[12] and TRIPOD-AI (Transparent Reporting of a Multivariable Prediction Model for Individual Prognosis or Diagnosis)[13].

STARD-AI is being coordinated by a global Project Team and Steering Committee consisting of clinician scientists, computer scientists, journal editors, EQUATOR Network representatives, reporting guideline developers, epidemiologists, statisticians, industry leaders, funders, health policy makers, legal experts and medical ethicists. In devising STARD-AI, we view that connecting all of these key stakeholders across the world is of the utmost importance.

Aim

This study aims to produce a novel AI centred diagnostic accuracy checklist (STARD-AI) which appropriately accounts for the specific considerations warranted in the reporting of AI diagnostic accuracy studies.

Focus of STARD-AI

The scope of STARD-AI 2021 is to address studies that use AI techniques to assess diagnostic accuracy (or clinical performance). Such studies compare test results between individuals (typically patients) with and without a target condition (or disease). Samples or images from study participants undergo assessment by a diagnostic technique which is designed to pick-up the target condition. This occurs alongside a concomitant reference standard or “gold-standard” test for the target condition in a defined timeframe. The diagnostic technique can account for either single or combined tests and typically includes (1) imaging data (e.g. CT scans), (2) pathological data (digitised specimen slide) or (3) reporting data (e.g. electronic health records or multi-omic spectra). STARD-AI 2021 also accounts for image segmentation and data delineation between a target condition and its absence (such as normal anatomy or health record results).

Estimates of clinical performance, or accuracy, are based on a comparison of the classification based on the test results with the classification by the reference standard, or gold standard, of the same patients. Alternatively, the reference standard can be the occurrence of an event within a defined timeframe.

STARD-AI was developed to guide the reporting of evaluations of the accuracy, or performance, of AI applications. If the emphasis of the study is on developing, validating, or updating a multivariable prediction model, the TRIPOD-AI reporting guidelines (Transparent Reporting of a multivariable prediction model for Individual Prognosis Or Diagnosis) may be more appropriate.

For peer review only

Methods

This protocol has been constructed in accordance with the EQUATOR Network (Enhancing the Quality and Transparency of Health Research) toolkit for developing reporting guidelines[14]. It has also greatly benefitted from the experience and expertise from Project Team and Steering Committee members who had previously led the STARD 2003[15], STARD 2015, STARD for Abstracts[16], SPIRIT-AI and CONSORT-AI initiatives respectively.

We are able to distil the development of the STARD-AI 2021 checklist into six stages. The overall goal of the STARD-AI initiative is to generate a list of minimally essential items, based upon the established STARD 2015 framework, that should be reported in all AI diagnostic accuracy studies. The items must assist the reader to appraise the completeness, applicability and potential for bias of the study findings.

Stage 1: Project organisation

A ten member STARD-AI Project Team was established in order to coordinate the guideline development process. The Project Team consists of the founder of STARD (PMB), the former United Kingdom Minister for Health and the current chair for the National Health Service Accelerated Access Collaborative (AD), members of the TRIPOD-AI core committee (GSC, LH, KGM), a senior software engineer (SS), directors of the EQUATOR Network (DM, GSC), the scientific content deputy editor for JAMA (RG) as well as 2 clinician scientists from Imperial College London (HA (supervisor), VS (doctoral research fellow)). The Project Team are responsible for identifying suitable members of the Steering Committee, candidate item generation, undertaking the online surveys for the modified Delphi consensus process, organising the consensus meeting, drafting the STARD-AI 2021 checklist and accompanying documents, coordinating the piloting the draft STARD-AI checklist as well as leading the dissemination process.

Further to the Project Team, a multidisciplinary STARD-AI Steering Committee was established in order to provide specialist guidance throughout the STARD-AI process. This committee consists of clinician scientists, computer scientists, journal editors, EQUATOR network directors, epidemiologists, statisticians, industry leaders, funders, health policy leaders, regulatory leaders, legal experts, patient representation experts and medical ethicists. These individuals were identified through their notable work with respect to (1) diagnostic accuracy research and its associated clinical translation, (2) applied artificial intelligence in healthcare as well as (3) notable contribution to other AI-centred EQUATOR Network registered initiatives, such as TRIPOD-AI, CONSORT-AI and SPIRIT-AI.

Prior to Stage 2, the STARD-AI project was registered with the EQUATOR Network.

Stage 2: Item generation

In order to generate a candidate list of items to enter the modified Delphi consensus process, the Project Team undertook a literature review, an extensive online scoping survey with an international panel of experts and a patient public involvement and engagement (PPIE) exercise.

a) Literature review:

In January 2020, a literature review of both academic and non-academic literature was undertaken. An electronic database search of Medical Literature Analysis and Retrieval System Online (MEDLINE) and Excerpta Medica database (EMBASE) was conducted through Ovid. Both Medical Subject Headings (MeSH) or EMBASE Subject Headings (Emtree) were used. Search results will be imported into Covidence (Covidence.org, Melbourne, Australia) for duplicate removal and study selection. Two individuals (VS/HA) individually screened study titles and abstracts for inclusion. Disagreements were resolved through discussion.

For peer review only

This process was augmented by non-systematic searches using traditional search engines for grey literature, social networking platforms as well as personal article collections highlighted by members of the Project Team. Titles and abstracts of shortlisted publications were screened by one of two reviewers (VS, HA) and potentially eligible publications were retrieved for full-text assessment. Extracted material were broadly classified into four categories by VS and HA; (1) general considerations regarding diagnostic accuracy studies and artificial intelligence, (2) evidence and statements suggesting modification to the STARD 2015 checklist, (3) evidence and statements suggesting additions to the STARD 2015 checklist and (4) evidence and statements suggesting the removal of specific items from the STARD 2015 checklist.

b) Online scoping survey:

In addition to this, in February 2020, the Project Team undertook an online survey with an international panel of experts (n=80) in order to identify potential further items or modifications that warrant consideration. This process generated over 2500 responses, which were analysed and classed into the aforementioned 4 broad categories.

c) Patient public involvement and engagement (PPIE) exercise:

Lastly, a focus group was conducted with patients and members of the public who had expressed an interest in participating in forums related to digital health and AI. The objective of these discussions was two-fold; (1) to further identify issues not uncovered during the literature review and expert survey and (2) to gain further understanding of the perceived importance of specific items raised thus far. These discussions were conducted remotely using Zoom (Zoom Video Communications, Inc., USA).

For peer review only

An expert facilitator led a discussion on the current use of AI in healthcare, on what the aims of STARD-AI were and what participants considered to be important items to capture during the study process. As stakeholder discussions were conducted virtually on Zoom, anonymised post-hoc discussion transcripts were maintained. Two investigators (VS, HA) independently identified common themes and sub-themes from the discussion, which were classed into the aforementioned 4 broad categories.

Having synthesised the findings of the literature review, the survey and the patient public involvement and engagement exercise, the Project Team, in collaboration with the Steering Committee, decided upon which items warrant consideration in the formal modified Delphi consensus process.

Stage 3: Modified Delphi consensus process

a) Study design and participants:

We will adopt a pragmatic modified Delphi consensus methodology. The Delphi consensus methodology is a well-established method[17] of obtaining a collective opinion from a group of experts through a series of questionnaires; each one refined based upon feedback from respondents on a previous version.

Participants are invited to join the STARD-AI Consensus Group on account of their expertise as clinician scientists, computer scientists, journal editors, EQUATOR Network representatives, reporting guideline developers, epidemiologists, statisticians, industry leaders (e.g., clinician scientists, computer scientists and product managers from health technological companies), funders, health policy makers, legal experts and medical ethicists. Invited experts will be provided with three weeks to respond to the initial invitation to participate. Those who accept the invitation will be invited to complete each round of the modified Delphi consensus process. Those who contribute to both online

For peer review only - http://bmjopen.bmj.com/site/about/guidelines.xhtml

rounds will be acknowledged by name as an author, within a group authorship model, in the publication that arises from this study.

In each round of the modified Delphi consensus process, participants will be asked to grade each candidate item using a 5-point Likert-like scale (1 – very important, 2 – important, 3 – moderately important, 4 – slightly important, 5 – not at all important). The threshold for consensus will be predefined at ≥80%. Items which achieve ≥80% ratings of 1 or 2 will be deemed to be essential for inclusion and will be put forward for discussion in the final round (round 3, which will occur in the form of a virtual teleconference meeting). Items which achieve ≥80% ratings of 4 or 5 will be deemed unimportant for inclusion and will be excluded. Items which did not reach this threshold of consensus will be put forward to the next round of the modified Delphi consensus process. In addition to rating items, participants will again be asked in a free-text format to suggest any other items that they consider to be potentially important to discuss in subsequent rounds.

In round 2, the survey will compose of items for which consensus was not achieved and any new items suggested in round 1. Next to each item, participants will be reminded of what rating they gave in the previous round. Additionally, the mean score given by the overall group in the previous round will be displayed for each item. Thus, participants will be able to revise their initial score with the additional knowledge of other participant responses. Following collection of round 2 responses, additional consensus items will be put forward for discussion during round 3 whilst negative consensus items will be excluded.

Any resulting non-consensus items from round 3 will again be put forward for voting in a final round, which will occur alongside the teleconference consensus meeting. Any final non-consensus items will then be resolved through discussion amongst those in virtual attendance at the consensus meeting.

For peer review only

b) Round 3; the consensus meetimg:

The consensus meeting (round 3) will consist of the STARD-AI Project Team and the STARD-AI Steering Committee. Given COVID-19 constraints, the meeting will be conducted virtually using Zoom (San Jose, United States of America). The primary objective is to develop a consensual draft version of STARD-AI checklist. As recommended in the COMET handbook, the nominal group technique, a highly-structured group interaction framework, will be utilised to aid this process[18,19]. Following a brief introduction and explanation of the purpose of the meeting by the facilitators (VS and HA), participants will discuss the inclusion and exclusion of candidate items. Participants will be asked to share any comments they have generated in a 'round robin' format until all contributions are exhausted. Participants will then be invited to discuss or seek further clarification about any of the ideas or comments produced. This discussion phase will be led by the facilitator (VS and HA) to ensure that the discussion will not be dominated by any one individual and be as neutral as possible[20].

c) Study conduct:

VS and HA will be the Delphi facilitators for the online rounds as well as the teleconference consensus meeting. They are responsible for the creation of the questionnaires, the invitations, the responses, the reminders, the analysis as well as the feedback for subsequent rounds.

The first two rounds of the modified Delphi consensus process will be conducted as online surveys using the DelphiManager software (version 4.0), which is developed and maintained by the COMET (Core Outcome Measures in Effectiveness Trials) initiative. Round 3 (the consensus meeting) will be carried using Zoom.

For peer review only

Stage 4: Development of the (1) checklist, (2) statement and (3) explanation and elaboration (E&E) document

Upon completion of the modified Delphi consensus process, the Project Team will draft the initial STARD-AI checklist and statement. The draft checklist and statement will be shared amongst the wider Steering Committee in order to discuss its content and therefore allowing the Steering Committee to suggest additions, subtractions or modifications as they see fit. This stage will also allow for harmonisation of key terms with the imminent TRIPOD-AI, in addition to the existing CONSORT-AI and SPIRIT-AI checklists.

Stage 5: Piloting amongst experts and non-experts

Upon completion of the first draft of the STARD-AI checklist, we intend to organise multiple rounds of piloting amongst expert and non-expert users (Pilot Group). The main aim of these piloting sessions is to identify items which are considered to be vague, ambiguous or perceived to be missing. We intend to undertake this process amongst radiology experts, pathology experts, computer scientists, expert statisticians, journal editorial boards, members of the global EQUATOR Network, key industry stakeholders as well as policy experts. Interviews amongst this Pilot Group will be undertaken in order to ensure that a granular level of feedback is attained for points of discussion. Experts and non-experts within the Pilot Group will be acknowledged by name as an author, within a group authorship model, in the publications that arise from this study.

In conjunction to this piloting process, the Project Team will also prepare the explanation and elaboration (E&E) document, to provide rationale for the included items along with examples of good reporting.

Stage 6: Finalisation, publication and post-publication activities

Following the piloting phase, the final proposed amendments to STARD-AI will be discussed amongst the Project Team and the Steering Committee. Once consensus has been reached through e-mail correspondence, the documents will be disseminated.

At this stage, a further discussion regarding the final strategy for dissemination and implementation of STARD-AI will occur amongst the Project Team and the Steering Committee. We strongly anticipate that the dissemination strategy will be principally tailored towards 5 groups of stakeholders; (a) academia, (b) policy, (c) guidelines and regulation, (d) industry and (e) patient representing bodies. Although a significant amount of material will cross over between stakeholders, creating stakeholder specific material is considered to be the most meaningful way of achieving impact.

a) <u>Academic stakeholders:</u>

We aim to publish the STARD-AI checklist, the accompanying statement and the E&E document in an open access format in a high-impact peer-reviewed journal. We will also share all relevant material through the EQUATOR website. In order to further complement this, we aim to create specialty-specific discourse regarding STARD-AI through focussed editorials in pertinent journals. These journal editors will also be actively encouraged to endorse STARD-AI as part of their broader editorial policy. Moreover, we will present STARD-AI at national and international scientific meetings. Translations of the guideline in various languages are actively encouraged in order to further broaden the scope of its impact. We encourage interested parties to contact the corresponding author for further information about the translation policies.

b) <u>Policy stakeholders:</u>

We aim to persuade governmental bodies to adopt the checklist as part of their policy assessments. This will involve presentations at national and international health policy summits (e.g., World

Innovation Summit for Health, NHS Accelerated Access Collaborative, National Institutes of Health). Furthermore, we will aim to integrate teaching about STARD-AI into national health policy educational programmes (the master's programme (MSc) for Health Policy at Imperial College London, the NHS Digital Academy, UK Research Innovation Centres of Excellence in AI in Digital Imaging).

c) Guidelines and regulatory stakeholders:

We aim to work alongside guidelines and regulatory bodies to adopt the checklist as part of their national health technology assessments. This will involve the United States Food and Drug Administration (FDA), the Medicines and Healthcare products Regulatory Agency (MHRA), The National Institute for Health and Care Excellence (NICE), the Horizon 2020 programme, the European Medicines Agency as well as the Consortia for Improving Medicine with Innovation and Technology (CIMIT).

d) Industry stakeholders:

We will present STARD-AI to a broad range of health technology companies (ranging from start-ups, small and medium-sized enterprises to multinational corporations) so that their product pipelines may accommodate for this.

e) Public and non-specific stakeholders:

Ensuring that the core material (STARD-AI checklist, statement and explanation and elaboration document) is available in an open access fashion, through a CC-BY license, is paramount to achieving general impact. In addition, we aim to publish articles in mainstream media and attain distribution through non-traditional means (e.g. social networking platforms, webinars, podcast episodes and blog posts).

Ethics

Ethical approval has been granted by the Joint Research Compliance Office at Imperial College London (SETREC reference number: 19IC5679).

Author Statement

Viknesh Sounderajah, Hutan Ashrafian, Robert Golub, Shravya Shetty, Jeffrey De Fauw, Lotty Hooft, Carl Moons, Gary Collins, David Moher, Patrick Bossuyt and Ara Darzi were involved in the planning and design of the study. Viknesh drafted the manuscript with all authors contributing to the writing.

Alan Karthikesalingam, Alastair Denniston, Bilal Mateen, Daniel Ting, Darren Treanor, Dominic King, Felix Greaves, Jonathan Godwin, Jonathan Pearson-Stuttard, Leanne Harling, Matthew McInnes, Nader Rifai, Nenad Tomasev, Pasha Normahani, Penny Whiting, Ravi Aggarwal, Sebastian Vollmer, Sheraz Markar, Trishan Panch and Xiaoxuan Liu are members of the STARD-AI Steering Committee. They are equally involved in the wider conduct and direction of the overall study. All of the authors edited the manuscript and provided critical appraisal.

All named authors approved the final draft of the manuscript.

For peer review only

References

1 Sounderajah V, Patel V, Varatharajan L, *et al.* Are disruptive innovations recognised in the healthcare literature? A systematic review. *BMJ Innov* 2020;:bmjinnov-2020-000424. doi:10.1136/bmjinnov-2020-000424

2 Topol EJ. High-performance medicine: the convergence of human and artificial intelligence. Nat. Med. 2019;**25**:44–56. doi:10.1038/s41591-018-0300-7

3 Benjamens S, Dhunnoo P, Meskó B. The state of artificial intelligence-based FDA-approved medical devices and algorithms: an online database. *npj Digit Med* 2020;**3**:118. doi:10.1038/s41746-020-00324-0

4 Williams BJ, Bottoms D, Treanor D. Future-proofing pathology: The case for clinical adoption of digital pathology. *J Clin Pathol* 2017;**70**:1010–8. doi:10.1136/jclinpath-2017-204644

5 Bossuyt PM, Reitsma JB, Bruns DE, *et al.* STARD 2015: An updated list of essential items for reporting diagnostic accuracy studies. *BMJ* 2015;**351**. doi:10.1136/bmj.h5527

6 Ochodo EA, Bossuyt PM. Reporting the Accuracy of Diagnostic Tests: The STARD Initiative 10 Years On. *Clin Chem* 2013;**59**:917–9. doi:10.1373/clinchem.2013.206516

7 Korevaar DA, Van Enst WA, Spijker R, *et al.* Reporting quality of diagnostic accuracy studies: A systematic review and meta-analysis of investigations on adherence to STARD. Evid. Based. Med. 2014;**19**:47–54. doi:10.1136/eb-2013-101637

8 Korevaar DA, Wang J, Van Enst WA, *et al.* Reporting diagnostic accuracy studies: Some improvements after 10 years of STARD. *Radiology* 2015;**274**:781–9. doi:10.1148/radiol.14141160

For peer review only

9 Sounderajah V, Ashrafian H, Aggarwal R, *et al.* Developing specific reporting guidelines for diagnostic accuracy studies assessing AI interventions: The STARD-AI Steering Group. Nat. Med. 2020;**26**:807–8. doi:10.1038/s41591-020-0941-1

10 The EQUATOR Network | Enhancing the QUAlity and Transparency Of Health Research. https://www.equator-network.org/ (accessed 26 Sep 2020).

11 Rivera SC, Liu X, Chan A-W, *et al.* Consensus statement Guidelines for clinical trial protocols for interventions involving artificial intelligence: the SPIRIT-AI extension The SPIRIT-AI and CONSORT-AI Working Group*, SPIRIT-AI and CONSORT-AI Steering Group and SPIRIT-AI and CONSORT-AI Consensus Group. *Nat Med* 2020;**26**:1351–63. doi:10.1038/s41591-020-1037-7

12 Liu X, Rivera SC. Consensus statement Reporting guidelines for clinical trial reports for interventions involving artificial intelligence: the CONSORT-AI extension6,13 ✉ and The SPIRIT-AI and CONSORT-AI Working Group*. *Nat Med 2020 269* 2020;**26**:1364–74. doi:10.1038/s41591-020-1034-x

13 Collins GS, Moons KGM. Reporting of artificial intelligence prediction models. Lancet. 2019;**393**:1577–9. doi:10.1016/S0140-6736(19)30037-6

14 Toolkits | The EQUATOR Network. https://www.equator-network.org/toolkits/ (accessed 26 Sep 2020).

15 Bossuyt PM, Reitsma JB, Bruns DE, *et al.* The STARD statement for reporting studies of diagnostic accuracy: explanation and elaboration. *Ann Intern Med* 2003;**138**. doi:10.7326/0003-4819-138-1-200301070-00012-w1

For peer review only

16 Cohen JF, Korevaar DA, Gatsonis CA, *et al.* STARD for Abstracts: Essential items for reporting diagnostic accuracy studies in journal or conference abstracts. *BMJ* 2017;**358**. doi:10.1136/bmj.j3751

17 Brown BB. Delphi Process: A Methodology Used for the Elicitation of Opinions of Experts. Published Online First: 1968.https://www.rand.org/pubs/papers/P3925.html (accessed 26 Sep 2020).

18 McMillan SS, King M, Tully MP. How to use the nominal group and Delphi techniques. *Int J Clin Pharm* 2016;**38**:655–62. doi:10.1007/s11096-016-0257-x

19 Williamson PR, Altman DG, Bagley H, *et al.* The COMET Handbook: version 1.0. *Trials* 2017;**18**:280. doi:10.1186/s13063-017-1978-4

20 Harvey N, Holmes CA. Nominal group technique: An effective method for obtaining group consensus. *Int J Nurs Pract* 2012;**18**:188–94. doi:10.1111/j.1440-172X.2012.02017.x

For peer review only

BMJ Open

Developing a Reporting Guideline for Artificial Intelligence Centred Diagnostic Test Accuracy Studies: The STARD-AI Protocol

Journal:	*BMJ Open*
Manuscript ID	bmjopen-2020-047709.R1
Article Type:	Protocol
Date Submitted by the Author:	04-May-2021

Complete List of Authors:	Sounderajah, Viknesh; Imperial College London, Department of Surgery and Cancer<br>Ashrafian, Hutan; Imperial College London, Department of Surgery and Cancer; Imperial College London, Department of Surgery and Cancer<br>Golub, Robert; Journal of the American Medical Association<br>Shetty, Shravya; Google Health<br>De Fauw, Jeffrey; DeepMind Technologies Ltd<br>Hooft, Lotty; University Medical Center Utrecht, University of Utrecht, Cochrane Netherlands<br>Moons, Karel; Julius Center for Health Sciences and Primary Care, Epidemiology<br>Collins, Gary; University of Oxford, Centre for Statistics in Medicine<br>Moher, David; Ottawa Hospital Research Institute, Ottawa Methods Centre<br>Bossuyt, Patrick M; Amsterdam University Medical Centres<br>Darzi, Ara; Imperial College London, Institute of Global Health Innovation<br>Karthikesalingam, Alan; Google Health<br>Denniston, Alastair; Queen Elizabeth Hospital Birmingham, UK<br>Mateen, Bilal Akhter; The Alan Turing Institute,<br>Ting, Daniel; Duke-NUS Medical School,<br>Treanor, Darren; University of Leeds<br>King, Dominic; Imperial College London, Centre for Health Policy<br>Greaves, Felix; Imperial College London, Department of Primary Care and Public Health<br>Godwin, Jonathan; DeepMind Technologies Ltd<br>Pearson-Stuttard, Jonathan; Imperial College London, PCPH<br>Harling, Leanne; Imperial College London, Department of Surgery and Cancer<br>McInnes, Matthew; University of Ottawa,<br>Rifai, Nader; Harvard Medical School,<br>Tomasev, Nenad; DeepMind Technologies Ltd<br>Normahani, Pasha; Imperial College London, Department of Surgery and Cancer<br>Whiting, Penny; University of Bristol, School of Social and Community Medicine<br>Aggarwal, Ravi; Imperial College London, Department of Surgery and Cancer<br>Vollmer, Sebastian; The Alan Turing Institute

	Markar, Sheraz; Imperial College London,<br>Panch, Trishan<br>Liu, Xiaoxuan; University of Birmingham
**Primary Subject Heading**:	Health informatics
Secondary Subject Heading:	Evidence based practice, Health policy
Keywords:	Protocols & guidelines < HEALTH SERVICES ADMINISTRATION & MANAGEMENT, Health informatics < BIOTECHNOLOGY & BIOINFORMATICS, Quality in health care < HEALTH SERVICES ADMINISTRATION & MANAGEMENT

SCHOLARONE™
Manuscripts

I, the Submitting Author has the right to grant and does grant on behalf of all authors of the Work (as defined in the below author licence), an exclusive licence and/or a non-exclusive licence for contributions from authors who are: i) UK Crown employees; ii) where BMJ has agreed a CC-BY licence shall apply, and/or iii) in accordance with the terms applicable for US Federal Government officers or employees acting as part of their official duties; on a worldwide, perpetual, irrevocable, royalty-free basis to BMJ Publishing Group Ltd ("BMJ") its licensees and where the relevant Journal is co-owned by BMJ to the co-owners of the Journal, to publish the Work in this journal and any other BMJ products and to exploit all rights, as set out in our licence.

The Submitting Author accepts and understands that any supply made under these terms is made by BMJ to the Submitting Author unless you are acting as an employee on behalf of your employer or a postgraduate student of an affiliated institution which is paying any applicable article publishing charge ("APC") for Open Access articles. Where the Submitting Author wishes to make the Work available on an Open Access basis (and intends to pay the relevant APC), the terms of reuse of such Open Access shall be governed by a Creative Commons licence – details of these licences and which Creative Commons licence will apply to this Work are set out in our licence referred to above.

Other than as permitted in any relevant BMJ Author's Self Archiving Policies, I confirm this Work has not been accepted for publication elsewhere, is not being considered for publication elsewhere and does not duplicate material already published. I confirm all authors consent to publication of this Work and authorise the granting of this licence.

For peer review only

Developing a Reporting Guideline for Artificial Intelligence Centred Diagnostic Test Accuracy Studies: The STARD-AI Protocol

Authors:

Viknesh Sounderajah[1,2], Hutan Ashrafian[1,2], Robert Golub[11], Shravya Shetty[6], Jeffrey De Fauw[3], Lotty Hooft[18], Karel Moons[18], Gary Collins[17], David Moher[12], Patrick Bossuyt[13] and Ara Darzi[1,2] on behalf of the STARD-AI Steering Committee (Alan Karthikesalingam[6], Alastair Denniston[4,14,15,16], Bilal Mateen[18], Daniel Ting[10], Darren Treanor[20], Dominic King[21], Felix Greaves[5], Jonathan Godwin[3], Jonathan Pearson-Stuttard[9], Leanne Harling[2], Matthew McInnes[7], Nader Rifai[22], Nenad Tomasev[3], Pasha Normahani[2], Penny Whiting[23], Ravi Aggarwal[1], Sebastian Vollmer[19], Sheraz Markar[2], Trishan Panch[8] and Xiaoxuan Liu[4,14,15,16])

Author Affiliations

[1] Institute of Global Health Innovation, Imperial College London, United Kingdom

[2] Department of Surgery and Cancer, Imperial College London, United Kingdom

[3] DeepMind, United Kingdom

[4] Institute of Inflammation and Ageing, College of Medical and Dental Sciences, University of Birmingham, United Kingdom

[5] The National Institute for Health and Care Excellence, United Kingdom

[6] Google Health

[7] Department of Radiology, University of Ottawa, Canada

[8] Division of Health Policy and Management, Harvard T.H. Chan School of Public Health, United States of America

[9] School of Public Health, Imperial College London, United Kingdom

[10] Singapore Eye Research Institute, Singapore National Eye Center, Singapore

[11] JAMA (Journal of the American Medical Association), United States of America

For peer review only

[12] Ottawa Hospital Research Institute, Canada

[13] Department of Clinical Epidemiology, Biostatistics and Bioinformatics, University of Amsterdam, The Netherlands

[14] University Hospitals Birmingham NHS Foundation Trust, Birmingham, United Kingdom

[15] Health Data Research UK, London, United Kingdom

[16] Clinical Epidemiology Program, Ottawa Hospital Research Institute, Canada

[17]Centre for Statistics in Medicine, Nuffield Department of Orthopaedics, Rheumatology and Musculoskeletal Sciences, University of Oxford, Oxford OX3 7LD, United Kingdom

[18]Julius Center for Health Sciences and Primary Care, and Cochrane Netherlands, University Medical Center Utrecht, Utrecht University, Utrecht, The Netherlands

[19] Alan Turing Institute, Kings Cross, United Kingdom

[20] Leeds Teaching Hospitals NHS Trust, University of Leeds, Leeds, United Kingdom

[21] Optum, Paddington, London, United Kingdom

[22] Department of Laboratory Medicine, Boston Children's Hospital, Harvard Medical School, Boston, Massachusetts, United States of America

[23] School of Social and Community Medicine, University of Bristol, Bristol, United Kingdom

Author disclosures:

The views and opinions expressed herein are those of the authors and do not necessarily reflect the views of their employers or funders.

Corresponding author:

Mr Hutan Ashrafian BSc (Hons) MBBS MRCS PhD MBA

Institute of Global Health Innovation, 10th Floor, Queen Elizabeth Queen Mother building, St Mary's Hospital Campus, Praed Street, London, United Kingdom, W2 1NY

Telephone Number: +447799871597

For peer review only

E-mail: hutan@imperial.ac.uk

Data Statement:

There is no data in this work.

Word count (main body):

3360

Study Status:

Stages 1 and 2 of this study has been completed. Stage 3 is underway (the study is currently between round 1 and round 2 of the modified Delphi consensus process).

For peer review only

Abstract

Introduction:

STARD was developed to improve the completeness and transparency of reporting in studies investigating diagnostic test accuracy. However, its current form, STARD 2015 does not address the issues and challenges raised by artificial intelligence (AI) centred interventions. As such, we propose an AI-specific version of the STARD checklist (STARD-AI), which focuses upon the reporting of AI diagnostic test accuracy studies. This paper describes the methods that will be used to develop STARD-AI.

Methods and analysis:

The development of the STARD-AI checklist can be distilled into six stages. (1) A project organisation phase has been undertaken, during which a Project Team and a Steering Committee were established. (2) An item generation process has been completed following a literature review, a patient and public involvement and engagement (PPIE) exercise and an online scoping survey of international experts. (3) A three-round modified Delphi consensus methodology is underway, which will culminate in a teleconference consensus meeting of experts. (4) Thereafter, the Project Team will draft the initial STARD-AI checklist and the accompanying documents. (5) A piloting phase amongst expert users will be undertaken to identify items which are either unclear or missing. This process, consisting of surveys and semi-structured interviews, will contribute towards the explanation and elaboration document. (6) Upon finalisation of the manuscripts, the group's efforts turn towards an organised dissemination and implementation strategy to maximise end-user adoption.

Ethics and dissemination:

Ethical approval has been granted by the Joint Research Compliance Office at Imperial College London (reference number: 19IC5679). A dissemination strategy will be aimed towards 5 groups of

For peer review only

stakeholders: (a) academia, (b) policy, (c) guidelines and regulation, (d) industry and (e) public and non-specific stakeholders. We anticipate that dissemination will take place in Q3 of 2021.

Key words:

Diagnostic accuracy, reporting guideline, artificial intelligence, STARD, transparency

Word count: 285/300

For peer review only

Article Summary

Strengths and limitations of this study:

- Gap: There are no specific reporting standards for artificial intelligence (AI) diagnostic test accuracy studies
- Solution: We are developing a specific set of reporting standards for AI diagnostic test accuracy studies; STARD-AI.
- Clinical implications: This will help key stakeholders to appraise quality and compare diagnostic test accuracy of AI models that are reported in scientific studies.
- Strengths: STARD-AI will be the product of an extensive evidence generation process that is led by multiple stakeholders (clinician scientists, computer scientists, journal editors, EQUATOR Network representatives, reporting guideline developers, epidemiologists, statisticians, industry leaders, funders, health policy makers, patients, legal experts, and medical ethicists).
- Limitations: Views of Delphi panellists may differ from those experts who decline participation.

only

Glossary

Project Team

This consists of the founder of STARD (PMB), the former United Kingdom Minister for Health and the current chair for the National Health Service Accelerated Access Collaborative (AD), members of the TRIPOD-AI group (GSC, KGM), a senior software engineer (SS), directors of the EQUATOR Network (DM, GSC), the scientific content deputy editor for JAMA (RG) as well as 2 clinician scientists from Imperial College London (HA, VS).

Steering Committee

This consists of clinician scientists, computer scientists, journal editors, EQUATOR Network representatives, epidemiologists, statisticians, industry leaders, funders, health policy makers, legal experts, and medical ethicists.

Consensus Group

This consists of experts who participated in the modified Delphi consensus process (stage 3) of the study.

Pilot Group

This consists of experts who participated in the pilot phase (Stage 5) of the study.

Checklist

A document listing the minimally essential items that should be reported in all diagnostic test accuracy studies centred around artificial intelligence centred index tests. This constitutes the core of the reporting guideline.

Statement

For peer review only

A document which provides the rationale underpinning the reporting guideline and describes the process of developing the associated documents.

Explanation and Elaboration (E&E)

A document which provides the rationale behind each item in the checklist alongside examples of good reporting.

Reporting guideline

The combination of the checklist, statement and E&E documents.

Artificial Intelligence (AI)

The science of developing computer systems which can perform tasks which normally require human intelligence.

Modified Delphi study

A research method that derives the collective opinions of a group through a staged consultation of surveys, questionnaires, or interviews, with an aim to reach consensus at the end.

For peer review only

Introduction

Artificial intelligence (AI) is commonly cited as an imminent disruptive innovation[1] within the health sector. If used successfully, AI has the potential to tackle (1) the high rate of avoidable medical errors, (2) workflow inefficiencies and (3) delivery inefficiencies associated with modern healthcare provision[2]. The majority of AI interventions that are close to translation are in the field of medical diagnostics[3]. In the current paradigm, diagnostic investigations require timely interpretation from an expert clinician in order to generate a diagnosis and to subsequently direct episodes of care. However, the recurring issue with the present system is that diagnostic services are inundated with large volumes of work, which often exceeds workforce capacity[4]; COVID-19 being an immediate case in point. In order to address this, diagnostic AI algorithms have positioned themselves as medical devices that may achieve diagnostic accuracy comparable to that of an expert clinician whilst concurrently alleviating health-resource use. Although this paradigm shift may seem imminent, it is crucial to note that much of the evidence supporting diagnostic algorithms has been disseminated in the absence of AI-specific reporting guidelines. Without this guidance, and in a relatively nascent area, key stakeholders are poorly placed to appraise quality and compare diagnostic accuracy between scientific studies.

The STARD (Standards for Reporting of Diagnostic Accuracy Studies) 2015 statement remains the most widely accepted set of reporting standards for diagnostic test accuracy studies[5]. STARD was developed to improve the completeness and transparency of studies investigating diagnostic test accuracy. It consists of a checklist of 30 items that authors are strongly encouraged to address when reporting their diagnostic test accuracy studies. It is endorsed by over 200 biomedical journals[6] and studies have shown that adherence to the STARD checklist leads to improved reporting of key study parameters[7,8].

However, in its current iteration, STARD 2015 is not designed to address the issues and challenges raised by AI-driven modalities. Issues include unclear methodological interpretation (e.g., data pre-processing steps, model development choices and the use of external validation datasets), the lack of standardized nomenclature (e.g., the varying definition of the term ‘validation’), as well as the use of unfamiliar outcome measures (e.g., Jaccard similarity coefficient and F-score). Until these issues are addressed, achieving comprehensive evaluations of these technologies and their potential translational benefits will remain limited.

In order to tackle these problems, we propose an AI-specific STARD guideline (STARD-AI) that aims to focus upon the reporting of AI diagnostic test accuracy studies[9]. This work is complementary to the other AI centred checklists listed in the EQUATOR (Enhancing Quality and Transparency of Health Research) Network program (www.equator-network.org)[10], such as SPIRIT-AI (Standard Protocol Items: Recommendations for Interventional Trials)[11], CONSORT-AI (Consolidated Standards of Reporting Trials)[12] and TRIPOD-AI (Transparent Reporting of a Multivariable Prediction Model for Individual Prognosis or Diagnosis)[13].

STARD-AI is being coordinated by a global Project Team and Steering Committee consisting of clinician scientists, computer scientists, journal editors, EQUATOR Network representatives, reporting guideline developers, epidemiologists, statisticians, industry leaders, funders, health policy makers, legal experts and medical ethicists.

Aim

This study aims to produce a specific reporting guideline (STARD-AI) for AI-centred diagnostic test accuracy studies.

Focus of STARD-AI

The focus of STARD-AI is to aid the comprehensive reporting of research that use AI techniques to assess diagnostic test accuracy and performance. This can account for either single or combined test data, which often consists of either (1) imaging data (e.g., CT scans), (2) pathological data (e.g. digitised specimen slide) or (3) reporting data (e.g. electronic health records). STARD-AI may also be used within studies which report upon image segmentation and other relevant data classification techniques. If the emphasis of the study is on either developing, validating or updating a multivariable prediction model which produces an individualised probability of developing a condition (e.g., time-to-event prediction), the TRIPOD-AI reporting guidelines (Transparent Reporting of a multivariable prediction model for Individual Prognosis Or Diagnosis) may be more appropriate.

Typically, diagnostic test accuracy studies compare test results between participants who are either with or without a target condition. Data from study participants undergo assessment by an index test, which is designed to identify a specific target condition. This process occurs alongside a concurrent reference standard for the target condition within a defined timeframe. Estimates of performance are typically based on a comparison between index test results and reference standard results from the same participant cohort. Alternatively, diagnostic performance can compare the performance of an index test against a reference standard determined through the incidence of an event within a defined timeframe.

A significant number of contemporary AI diagnostic studies include information related to both the development and testing (validation) of AI centred index tests. In order to accommodate and improve upon this practice, STARD-AI will propose items related to AI index test development and validation as part of the consensus process. Other key topics for consideration within this study include, but are not limited to, the following: (1) data pre-processing methods, (2) AI index test development methods (e.g., dataset partition, model calibration, stopping criteria when training, use of external validation sets), (3) fairness metrics, (5) non-standard performance metrics, (5) explainability and (6) human-AI

For peer review only

index test interaction. As noted in the methods section, the inclusion of specific items related to these issues is reliant upon consensus that is achieved through a transparent and fair evidence generation process.

For peer review only

Methods

This protocol has been constructed in accordance with the EQUATOR Network (Enhancing the Quality and Transparency of Health Research) toolkit for developing reporting guidelines[14]. It has also greatly benefitted from the experience and expertise from Project Team and Steering Committee members who had previously led the STARD 2003[15], STARD 2015, STARD for Abstracts[16], SPIRIT-AI and CONSORT-AI initiatives respectively.

We can distil the development of the STARD-AI checklist into six stages. The overall goal of the STARD-AI initiative is to generate a list of minimally essential items, based upon the established STARD 2015 framework, that should be reported in all AI diagnostic test accuracy studies. The items must assist the reader to appraise the completeness, applicability, and potential for bias of the study findings.

Stage 1: Project organisation

A nine member STARD-AI Project Team was established to coordinate the reporting guideline development process. The Project Team consists of the founder of STARD (PMB), the former United Kingdom Minister for Health and the current chair for the National Health Service Accelerated Access Collaborative (AD), members of the TRIPOD-AI core committee (GSC, KGM), a senior software engineer (SS), directors of the EQUATOR Network (DM, GSC), the scientific content deputy editor for JAMA (RG) as well as 2 clinician scientists from Imperial College London (HA, VS). The Project Team are responsible for identifying suitable members of the Steering Committee, candidate item generation, undertaking the online surveys for the modified Delphi consensus process, organising the consensus meeting, drafting the STARD-AI checklist and accompanying documents, piloting the draft STARD-AI checklist as well as leading upon the dissemination process.

Further to the Project Team, a multidisciplinary STARD-AI Steering Committee was established to provide specialist guidance throughout. This committee consists of clinician scientists, computer scientists, journal editors, EQUATOR network directors, epidemiologists, statisticians, industry leaders, funders, health policy leaders, regulatory leaders, legal experts, patient representation experts and medical ethicists. These individuals were identified through their notable work with respect to (1) diagnostic accuracy research and its clinical translation, (2) applied artificial intelligence in healthcare as well as (3) notable contribution to other AI-centred EQUATOR Network registered initiatives, such as TRIPOD-AI, CONSORT-AI and SPIRIT-AI.

Prior to Stage 2, the STARD-AI project was registered with the EQUATOR Network.

Stage 2: Item generation

In order to generate a candidate list of items to enter the modified Delphi consensus process, the Project Team undertook a literature review, an online scoping survey with an international panel of experts and a patient public involvement and engagement (PPIE) exercise.

a) Literature review:

In January 2020, a literature review of both academic and non-academic literature was undertaken. An electronic database search of Medical Literature Analysis and Retrieval System Online (MEDLINE) and Excerpta Medica database (EMBASE) was conducted through Ovid. Both Medical Subject Headings (MeSH) or EMBASE Subject Headings (Emtree) were used. Search results were imported into Covidence (Covidence.org, Melbourne, Australia) for duplicate removal and study selection. Two individuals (VS,HA) individually screened study titles and abstracts for inclusion. Disagreements were resolved through discussion.

For peer review only - http://bmjopen.bmj.com/site/about/guidelines.xhtml

This process was augmented by non-systematic searches using grey literature, social networking platforms as well as personal article collections highlighted by members of the Project Team. Titles and abstracts of shortlisted publications were screened by one of two reviewers (VS, HA) and potentially eligible publications were retrieved for full-text assessment. Extracted material were broadly classified into four categories: (1) general considerations regarding diagnostic accuracy studies and artificial intelligence, (2) evidence and statements suggesting modification to existing STARD 2015 items, (3) evidence and statements suggesting additions to the STARD 2015 checklist and (4) evidence and statements suggesting the removal of specific items from the STARD 2015 checklist.

b) Online scoping survey:

In addition to this, in February 2020, the Project Team undertook an online survey with an international panel of 80 experts in order to identify potential further items or modifications that warrant consideration. Written participant consent was attained as part of this process. This process generated over 2500 responses, which were analysed and classed into the aforementioned 4 broad categories.

c) Patient public involvement and engagement (PPIE) exercise:

Lastly, a focus group was conducted with patients and members of the public who had expressed an interest in participating in forums related to digital health and AI. Written participant consent was attained as part of this process. The objective of these discussions was two-fold; (1) to further identify issues not uncovered during the literature review and expert survey and (2) to gain further understanding of the perceived importance of specific items raised thus far. These discussions were conducted remotely using Zoom (Zoom Video Communications, Inc., USA).

For peer review only - http://bmjopen.bmj.com/site/about/guidelines.xhtml

An expert facilitator led a discussion on the current use of AI in healthcare, on what the aims of STARD-AI were and what participants considered to be important items to capture during the study process. As stakeholder discussions were conducted virtually on Zoom, anonymised post-hoc discussion transcripts were maintained. Two investigators (VS, HA) independently identified common themes and sub-themes from the discussion, which were classed into the aforementioned 4 broad categories.

Having synthesised the findings of the literature review, the survey and the patient public involvement and engagement exercise, the Project Team, in collaboration with the Steering Committee, decided upon which items warranted consideration in the formal modified Delphi consensus process.

Stage 3: Modified Delphi consensus process (ongoing)

a) Study design and participants:

This study has adopted a pragmatic modified Delphi consensus methodology. The Delphi consensus methodology is a well-established method[17] of obtaining a collective opinion from a group of experts through a series of questionnaires; each one refined based upon feedback from respondents.

Participants were invited to join the STARD-AI Consensus Group on account of their expertise as clinician scientists, computer scientists, journal editors, EQUATOR Network representatives, reporting guideline developers, epidemiologists, statisticians, industry leaders (e.g., clinician scientists, computer scientists and product managers from health technological companies), funders, health policy makers, legal experts and medical ethicists. These experts were shortlisted through two principle means; either through the professional networks of members of the STARD-AI Project Team and Steering Committee or through recognition, critical involvement and achievements in a field that is related to diagnostic AI systems in the health sector (e.g., authorship of seminal academic

For peer review only

publications, key thought leaders, clinicians involved in prominent AI translational work and health policy directors, amongst others). Moreover, ensuring fair representation across geographies and demographics was a pertinent consideration during recruitment. Shortlisted participants were mutually agreed upon by the Project Team members.

Following this, invited experts were provided with three weeks to respond to the initial invitation to participate. Written participant consent was attained as part of this process. Those who accepted the invitation were invited to complete each round of the modified Delphi consensus process. Those who contribute to both online rounds will be acknowledged by name as an author, within a group authorship model, in the publication that arises from this study.

In each round of the modified Delphi consensus process, participants are asked to grade each candidate item using a 5-point Likert-like scale (1 – very important, 2 – important, 3 – moderately important, 4 – slightly important, 5 – not at all important). The threshold for consensus is predefined at ≥75%. Items which achieve ≥75% ratings of 1 or 2 are deemed to be essential for inclusion and are put forward for discussion in the final round (round 3, which will occur in the form of a virtual teleconference meeting). Items which achieve ≥75% ratings of 4 or 5 are deemed unimportant for inclusion and are excluded. Items which do not reach this threshold of consensus are put forward to the next round of the modified Delphi consensus process. In addition to rating items, participants are asked in a free-text format to suggest any other items that they consider to be important to discuss in subsequent rounds.

In round 2, the survey will compose of (1) items for which consensus was not achieved in round 1 and (2) any new items suggested as part of round 1 feedback. Next to each item, participants will be reminded of what rating they gave in the previous round. Additionally, the mean score given by the overall group in the previous round will be displayed for each item. Thus, participants will be able to

For peer review only

revise their initial score with the additional knowledge of peer responses. Following the collection of round 2 responses, additional items which achieve consensus as ‘important’ will be put forward for discussion during round 3. Those items that achieve consensus as ‘unimportant’ are excluded. Lastly, any non-consensus items from round 2 will be resolved through discussion amongst those in virtual attendance at the consensus meeting (round 3).

b) Round 3; the consensus meeting:

The consensus meeting (round 3) will consist of the STARD-AI Project Team and the STARD-AI Steering Committee. Given COVID-19 constraints, the meeting will be conducted virtually using Zoom. The primary objective is to develop a draft version of the STARD-AI checklist. As recommended in the COMET handbook, the nominal group technique, a highly-structured group interaction framework, will be utilised to aid this process[18,19]. Following a brief introduction and explanation of the purpose of the meeting by the facilitators (VS, HA), participants will discuss the inclusion and exclusion of candidate items. Participants will be asked to share any comments they have generated in a 'round robin' format until all contributions are exhausted. Participants will then be invited to discuss or seek further clarification about any of the ideas or comments produced. This discussion phase will be led by facilitators (VS, HA) to ensure that the discussion will not be dominated by any one individual and will be as neutral as possible[20].

c) Study conduct:

VS and HA are the Delphi facilitators for the online survey rounds as well as the teleconference consensus meeting. They are responsible for the creation of the questionnaires, the invitations, the responses, the reminders, the analysis as well as the feedback for subsequent rounds.

For peer review only

The first two rounds of the modified Delphi consensus process are conducted as online surveys using the DelphiManager software (version 4.0), which is developed and maintained by the COMET (Core Outcome Measures in Effectiveness Trials) initiative. Round 3 (the consensus meeting) will be carried using Zoom.

Stage 4: Development of the (1) checklist, (2) statement and (3) explanation and elaboration (E&E) document

Upon completion of the modified Delphi consensus process, the Project Team will draft the initial STARD-AI checklist and statement. The draft checklist and statement will be shared amongst the wider Steering Committee in order to discuss its content and therefore allow the Steering Committee to suggest additions, subtractions or modifications as they see fit. This stage will also allow for harmonisation of key terms with the imminent TRIPOD-AI, in addition to the existing CONSORT-AI and SPIRIT-AI checklists.

Stage 5: Piloting phase

Upon completion of the first draft of the STARD-AI checklist, we intend to organise a piloting phase amongst expert users (Pilot Group). The main aim of these piloting sessions is to identify items which are considered to be vague, unnecessary or missing. We intend to undertake this process amongst radiology experts, pathology experts, computer scientists, expert statisticians, journal editorial boards, members of the global EQUATOR Network, key industry stakeholders as well as policy experts. Much like stage 3, these experts are shortlisted through two principle means; either through the professional networks of members of the STARD-AI Project Team and Steering Committee or through either (1) involvement in teams that have led diagnostic AI studies or (2) work as peer reviewers or editorial board members for journals that publish diagnostic AI studies. Experts are mutually agreed upon by the Project Team members and Steering Committee. Feedback will be captured through

y only

surveys and a series of semi-structured interviews. This approach allows for the capture of broad issues through surveys, which form themes that can be further explored in detail during semi-structured interviews. Anonymised feedback from the interviews will be transcribed to allow for thematic analysis so that recurring trends are appropriately identified and presented back to the Project Team and Steering Committee for discussion. Experts within the Pilot Group will be acknowledged by name as an author, within a group authorship model, in the publications that arise from this study.

In conjunction to this piloting process, the Project Team will also prepare the explanation and elaboration (E&E) document to provide rationale for the included items alongside examples of good reporting.

Stage 6: Finalisation, publication, and post-publication activities

Following the piloting phase, the final proposed amendments to STARD-AI will be discussed amongst the Project Team and the Steering Committee. Once consensus has been reached through e-mail correspondence, the checklist and accompanying documents will be disseminated.

The dissemination strategy will be principally tailored towards 5 groups of stakeholders; (a) academia, (b) policy, (c) guidelines and regulation, (d) industry and (e) patient representing bodies. Although a significant amount of material will cross over between stakeholders, creating specific material is considered to be the most meaningful way of achieving impact.

We aim to publish the STARD-AI checklist, the accompanying statement and the E&E document in an open access format (through a CC-BY license). In order to further complement this, we aim to create specialty-specific discourse regarding STARD-AI through focussed editorials in pertinent journals. These journal editors will also be actively encouraged to endorse STARD-AI as part of their broader

For peer review only - http://bmjopen.bmj.com/site/about/guidelines.xhtml

editorial policy. Moreover, we will present STARD-AI at national and international scientific meetings. Translations of the guideline in various languages are actively encouraged (available on the EQUATOR network) in order to further broaden the scope of its impact. We encourage interested parties to contact the corresponding author for further information about the translation policies.

In addition to this, we aim to persuade governmental bodies to adopt the checklist as part of their policy assessments. This will involve presentations at national and international health policy summits (e.g., World Innovation Summit for Health and NHS Accelerated Access Collaborative meetings). Furthermore, we will aim to integrate teaching about STARD-AI into national health policy educational programmes through pre-existing collaborations with academic institutions, NHS Digital Academy and NHSX.

Concurrent to this workstream will be our work with guidelines and regulatory bodies so that they may account for STARD-AI as part of their national health technology assessments. This will involve the United States Food and Drug Administration (FDA), the Medicines and Healthcare products Regulatory Agency (MHRA) and The National Institute for Health and Care Excellence (NICE) amongst others.

Lastly, we will present STARD-AI to a broad range of health technology companies so that their product pipelines may accommodate for this downstream mode of assessment.

Conclusion:

STARD-AI will serve as the first global-consensus achieved guidance for the reporting of AI centred diagnostic accuracy studies. Through a clear multi-stakeholder dissemination policy, we hope that STARD-AI can significantly contribute towards minimising research waste as well as serving as an

instrument that assists the streamlined translation of these nascent technologies. We anticipate that STARD-AI will be published in Q3 2021.

For peer review only

Ethics

Ethical approval has been granted by the Joint Research Compliance Office at Imperial College London (SETREC reference number: 19IC5679).

Author Statement

Viknesh Sounderajah, Hutan Ashrafian, Robert Golub, Shravya Shetty, Jeffrey De Fauw, Lotty Hooft, Karel Moons, Gary Collins, David Moher, Patrick Bossuyt and Ara Darzi were involved in the planning and design of the study. Viknesh drafted the manuscript with all authors contributing to the writing.

Alan Karthikesalingam, Alastair Denniston, Bilal Mateen, Daniel Ting, Darren Treanor, Dominic King, Felix Greaves, Jonathan Godwin, Jonathan Pearson-Stuttard, Leanne Harling, Matthew McInnes, Nader Rifai, Nenad Tomasev, Pasha Normahani, Penny Whiting, Ravi Aggarwal, Sebastian Vollmer, Sheraz Markar, Trishan Panch and Xiaoxuan Liu are members of the STARD-AI Steering Committee. They are equally involved in the wider conduct and direction of the overall study. All of the authors edited the manuscript and provided critical appraisal.

All named authors approved the final draft of the manuscript.

Competing Interests

There are no competing interests for any author.

Funding

Infrastructure support for this research was provided by the NIHR Imperial Biomedical Research Centre (BRC).

For peer review only

For peer review only - http://bmjopen.bmj.com/site/about/guidelines.xhtml

GSC is supported by the NIHR Oxford Biomedical Research Centre and Cancer Research UK (programme grant: C49297/A27294).

DT is funded by National Pathology Imaging Co-operative, NPIC (Project no. 104687) is supported by a £50m investment from the Data to Early Diagnosis and Precision Medicine strand of the government's Industrial Strategy Challenge Fund, managed and delivered by UK Research and Innovation (UKRI).

FG is supported by the National Institute for Health Research Applied Research Collaboration Northwest London

For peer review only

References

1 Sounderajah V, Patel V, Varatharajan L, *et al.* Are disruptive innovations recognised in the healthcare literature? A systematic review. *BMJ Innov* 2020;:bmjinnov-2020-000424. doi:10.1136/bmjinnov-2020-000424

2 Topol EJ. High-performance medicine: the convergence of human and artificial intelligence. Nat. Med. 2019;**25**:44–56. doi:10.1038/s41591-018-0300-7

3 Benjamens S, Dhunnoo P, Meskó B. The state of artificial intelligence-based FDA-approved medical devices and algorithms: an online database. *npj Digit Med* 2020;**3**:118. doi:10.1038/s41746-020-00324-0

4 Williams BJ, Bottoms D, Treanor D. Future-proofing pathology: The case for clinical adoption of digital pathology. *J Clin Pathol* 2017;**70**:1010–8. doi:10.1136/jclinpath-2017-204644

5 Bossuyt PM, Reitsma JB, Bruns DE, *et al.* STARD 2015: An updated list of essential items for reporting diagnostic accuracy studies. *BMJ* 2015;**351**. doi:10.1136/bmj.h5527

6 Ochodo EA, Bossuyt PM. Reporting the Accuracy of Diagnostic Tests: The STARD Initiative 10 Years On. *Clin Chem* 2013;**59**:917–9. doi:10.1373/clinchem.2013.206516

7 Korevaar DA, Van Enst WA, Spijker R, *et al.* Reporting quality of diagnostic accuracy studies: A systematic review and meta-analysis of investigations on adherence to STARD. Evid. Based. Med. 2014;**19**:47–54. doi:10.1136/eb-2013-101637

8 Korevaar DA, Wang J, Van Enst WA, *et al.* Reporting diagnostic accuracy studies: Some improvements after 10 years of STARD. *Radiology* 2015;**274**:781–9. doi:10.1148/radiol.14141160

For peer review only

9 Sounderajah V, Ashrafian H, Aggarwal R, *et al.* Developing specific reporting guidelines for diagnostic accuracy studies assessing AI interventions: The STARD-AI Steering Group. Nat. Med. 2020;**26**:807–8. doi:10.1038/s41591-020-0941-1

10 The EQUATOR Network | Enhancing the QUAlity and Transparency Of Health Research. https://www.equator-network.org/ (accessed 26 Sep 2020).

11 Rivera SC, Liu X, Chan A-W, *et al.* Consensus statement Guidelines for clinical trial protocols for interventions involving artificial intelligence: the SPIRIT-AI extension The SPIRIT-AI and CONSORT-AI Working Group*, SPIRIT-AI and CONSORT-AI Steering Group and SPIRIT-AI and CONSORT-AI Consensus Group. *Nat Med* 2020;**26**:1351–63. doi:10.1038/s41591-020-1037-7

12 Liu X, Rivera SC. Consensus statement Reporting guidelines for clinical trial reports for interventions involving artificial intelligence: the CONSORT-AI extension6,13 ✉ and The SPIRIT-AI and CONSORT-AI Working Group*. *Nat Med 2020 269* 2020;**26**:1364–74. doi:10.1038/s41591-020-1034-x

13 Collins GS, Moons KGM. Reporting of artificial intelligence prediction models. Lancet. 2019;**393**:1577–9. doi:10.1016/S0140-6736(19)30037-6

14 Toolkits | The EQUATOR Network. https://www.equator-network.org/toolkits/ (accessed 26 Sep 2020).

15 Bossuyt PM, Reitsma JB, Bruns DE, *et al.* The STARD statement for reporting studies of diagnostic accuracy: explanation and elaboration. *Ann Intern Med* 2003;**138**. doi:10.7326/0003-4819-138-1-200301070-00012-w1

For peer review only - http://bmjopen.bmj.com/site/about/guidelines.xhtml

537 16 Cohen JF, Korevaar DA, Gatsonis CA, *et al.* STARD for Abstracts: Essential items for reporting diagnostic accuracy studies in journal or conference abstracts. *BMJ* 2017;**358**. doi:10.1136/bmj.j3751

540 17 Brown BB. Delphi Process: A Methodology Used for the Elicitation of Opinions of Experts. Published Online First: 1968.https://www.rand.org/pubs/papers/P3925.html (accessed 26 Sep 2020).

543 18 McMillan SS, King M, Tully MP. How to use the nominal group and Delphi techniques. *Int J Clin Pharm* 2016;**38**:655–62. doi:10.1007/s11096-016-0257-x

545 19 Williamson PR, Altman DG, Bagley H, *et al.* The COMET Handbook: version 1.0. *Trials* 2017;**18**:280. doi:10.1186/s13063-017-1978-4

547 20 Harvey N, Holmes CA. Nominal group technique: An effective method for obtaining group consensus. *Int J Nurs Pract* 2012;**18**:188–94. doi:10.1111/j.1440-172X.2012.02017.x

549

550

For peer review only
